# The Effects of Benralizumab on Lung Volumes and Airway Resistance in Severe Eosinophilic Asthma: A Real-World Study

**DOI:** 10.7759/cureus.52452

**Published:** 2024-01-17

**Authors:** António Madeira Gerardo, Carolina da Silva Alves, Margarida Gomes, Cecília Pardal, Anna Sokolova, Hedi Liberato, Ana Mendes, Fernanda S Tonin, Filipa Duarte-Ramos, Carlos Lopes

**Affiliations:** 1 Pulmonology, Hospital Professor Doutor Fernando Fonseca, Lisboa, PRT; 2 Allergy and Immunology, Hospital de Santa Maria, Unidade Multidisciplinar de Asma Grave, Lisboa, PRT; 3 Health and Technology Research Center, Escola Superior de Tecnologia da Saúde de Lisboa (ESTeSL) Instituto Politécnico de Lisboa (IPL), Lisbon, PRT; 4 Pharmacy, University of Lisbon, Lisbon, PRT; 5 Pulmonology, Hospital de Santa Maria, Unidade Multidisciplinar de Asma Grave, Lisboa, PRT

**Keywords:** respiratory function, anti-il5, benralizumab, t2 inflammation, severe eosinophilic asthma

## Abstract

Introduction: Add-on biological monoclonal antibodies such as benralizumab (anti-IL-5Ra) are recommended by international guidelines to reduce exacerbations in severe eosinophilic asthma (SEA). However, few studies have assessed the impact of these therapies on lung function-related outcomes. Our goal was to evaluate the effectiveness of benralizumab on lung function, including lung volumes and airway resistance, in SEA patients in Portugal.

Methods: This was a real-world, observational, prospective, multicentric study including adult patients diagnosed with SEA (January-June 2023). Spirometry and plethysmography were performed at baseline (T0) and after six months of treatment (T6) with benralizumab to assess: total lung capacity (TLC), residual volume (RV), forced expiratory volume in one second (FEV_1_), forced vital capacity (FVC), mean forced expiratory flow between 25% and 75% of FVC (mFEF-25/75), intrathoracic gas volume (ITGV), and respiratory airway resistance (Raw). Descriptive statistics (with categorical variables described as frequencies and continuous values as mean and standard deviation (SD)) and paired t-test and Cohen’s d effect size were calculated (analyses performed in StataCorp v.15.1; StataCorp LLC, TX, USA).

Results: Overall, 30 SEA patients were evaluated, mostly women (n=18, 60.0%), with atopy (n=22, 73.3%), a mean age of 58.4 years (SD 11.7), and assisted by pulmonology (n=19, 63.3%) or immunology-allergology (n=11, 36.7%) services. Mean eosinophilia at baseline was 1103.57 cells/mcL (SD 604.88; minimum-maximum 460-2400); after the use of benralizumab, the count dropped to zero. After six months of treatment, a significant increase (p<0.0001) in FVC (15.3%), FEV_1_ (22.6%), and mFEF-25/75 (17.7%) were observed from baseline (Cohen’s d between 0.78 and 1.11). ITGV, RV, RV/TLC, and Raw significantly decreased (p<0.0001) during the study period (-17.3%, -29.7%, -8.9%, and -100.6%, respectively) (Cohen’s d between -0.79 and -1.06). No differences in TLC were obtained (p=0.173). No differences between sexes were observed for any measure. Patients with more significant eosinophilia (>900 cells/mcL count; n=15) presented better responses in FEV_1 _(p=0.001) and mFEF-25/75 (p=0.007).

Conclusions: A notable eosinophil depletion with add-on benralizumab led to significant improvements in SEA patients’ respiratory function (static lung volumes and airway resistance) in real-world settings after six months. The significant deflating effect of benralizumab on patients’ hyperinflated lungs led to enhanced expiratory flow (increased FEV_1_ and mFEF-25/75) and air trapping (decreased RV/TLC), suggesting this antibody improves bronchial obstruction, lung hyperinflation, and airway resistance. Further studies in a larger population are required to confirm these findings.

## Introduction

Asthma, a chronic and heterogeneous inflammatory disease, has an overall prevalence of 10.5% (95% CI 9.5-11.6) in Portugal. It is characterized by airflow limitations and airway changes, manifesting in various forms (i.e., phenotypes) due to complex underlying biological mechanisms (i.e., endotypes) [1-3]. Regardless of being allergic or not, most patients present with bronchial eosinophilia, recurrent disease exacerbations, and severe airflow obstruction, often accompanied by increased sputum/blood eosinophil levels [2,4].

Severe asthma, defined as uncontrolled grades 4-5 according to the Global Initiative for Asthma and affecting 5-10% of patients [5,6], is typically marked by predominant eosinophilic inflammation. This inflammation is triggered by type-2 cellular responses, involving T helper 2 lymphocytes and group 2 innate lymphoid cells. The cytokines produced by these cells, including IL-4, 5, 9, and 13, lead to IgE synthesis, eosinophilic inflammation, mast cell development, and bronchial hyperresponsiveness. Eosinophilic phenotypes, often associated with atopy, are characterized by IgE overproduction [7-9]. Although not highly prevalent, these cases contribute significantly to the overall disease burden and asthma-related costs, accounting for 50-60% of the total costs, especially when the condition is uncontrolled [10,11].

Most allergic eosinophilic patients achieve good asthma control with inhaled corticosteroids (ICS). However, a small percentage experience therapeutic failure, even with both ICS and oral corticosteroids (OCS), due to mechanisms such as IL-5 and IL-13 oversecretion, histone deacetylase down-regulation, or glucocorticoid receptor impairment [12-14]. Current guidelines recommend add-on biological therapy using monoclonal antibodies like omalizumab (anti-IgE), mepolizumab or reslizumab (anti-IL-5), benralizumab (IL-5 receptor blocker), and dupilumab (dual IL-4/IL-13 receptor antagonist) to improve control of difficult-to-treat asthma [6,15-18].

Benralizumab, a humanized IgG1k monoclonal antibody, blocks the α subunit of the IL-5 receptor (IL-5Rα), preventing IL-5 binding and inhibiting eosinophil differentiation and maturation. It also triggers eosinophil apoptosis through antibody-dependent cell-mediated cytotoxicity [19]. These mechanisms have been shown to lead to significant clinical and functional improvements in both atopic and non-atopic severe eosinophilic asthma (SEA) patients. Evidence from various studies, including the phase III multicentric randomized SIROCCO and CALIMA trials, indicates that benralizumab can reduce annual rates of asthma exacerbations, improve asthma symptom control, and alleviate airway obstruction [20-24].

The therapeutic effects of benralizumab are interconnected with the direct relationship between disease exacerbations and bronchial obstruction. Recurrent exacerbations of severe asthma can accelerate the progressive decline of lung function due to worsening airway inflammation and remodeling [13]. Severe disease often results in significant air trapping and lung hyperinflation, marked by an increase in residual volume (RV), intrathoracic gas volume (ITGV), and total lung capacity (TLC), while decreasing inspiratory capacity. However, few studies to date, including the phase III SOLANA trial, have assessed lung function-related outcomes after the use of add-on biologics in SEA, with none performed in Portugal [13,25].

Therefore, our study aimed to evaluate the effectiveness of benralizumab on lung function, including lung volumes and airway resistance, in SEA patients in real-world practice in Portugal.

## Materials and methods

Study design, setting, and sample

This was a real-world, observational, prospective, and multicentric study (Hospital Santa Maria and Hospital Professor Doutor Fernando Fonseca, Portugal) describing a cohort of adult patients diagnosed with SEA and identified for this study between January and June 2023. Patients were followed for six months after treatment initiation with benralizumab (30 mg by subcutaneous injection every four weeks for the first three doses and then every eight weeks thereafter) as add-on therapy.

Patients were classified as SEA based on (1) the presence of type 2 eosinophilic phenotype defined as an eosinophil count of at least 150 cells/microliter in the past six weeks OR an eosinophil count of at least at least 300 cells/microliter in the past year; (2) patient having uncontrolled asthma defined as any of the following: two or more exacerbations in the past 12 months requiring corticosteroids for three days or more, one or more asthma exacerbation(s) leading to hospitalization in the past 12 months, asthma control test consistently less than 20 over the past 12 months, and dependence on daily oral corticosteroids for asthma control; (3) patient having uncontrolled asthma despite good adherence to a regimen containing a high dose ICS and at least one additional asthma controller medication OR daily oral corticosteroids. Patients were excluded if any of the following criteria were observed: patients under 18 years of age, patients previously treated with other biological agents, patients with exacerbations in the last eight weeks before undergoing spirometry and plethysmography, and patients with other obstructive pathologies.

Procedures followed standards for scientific research and were performed according to the Declaration of Helsinki. The Ethics Committee of the Hospital Professor Doutor Fernando Fonseca approved the study (approval number: 39/2023). Due to the non-interventional nature of this study, no informed consent forms were necessary.

Data collection and measurements

The following data was manually collected by the investigators from patients’ electronic medical records: demographic information (age, gender, date of diagnosis, follow-up service), disease characteristics (atopy, eosinophilia count), and treatment initiation with benralizumab. Pre-bronchodilation spirometry and plethysmography were performed at baseline (T0) and after six months of treatment (T6) with benralizumab to assess: TLC, RV, forced expiratory volume in one second (FEV_1_), forced vital capacity (FVC), mean forced expiratory flow between 25% and 75% of FVC (mFEF-25/75), ITGV, and Raw.

Data analysis

Descriptive statistics were used to summarize the data, with absolute and relative frequencies to describe categorical variables and mean and standard deviation (SD) for continuous values. A paired t-test and Cohen’s d effect-size measure were calculated to compare the means of two measurements taken from the same individual and the standardized mean difference between groups (i.e., pre/post analysis) (d=0.2 small, d=0.5 medium, d=0.8 large effect sizes). Subgroup analyses considering patients’ sex, presence of atopy, and eosinophilia severity (poorer status considered eosinophil count >900 cells/μL) were also performed.

Results were synthesized using tables and figures, including absolute differences and differences in percentage points (pp). Analyzes were performed in Stata v.15.1 (StataCorp, 2017. Stata Statistical Software: Release 15. College Station, TX: StataCorp LLC) with p-values below 5% considered statistically significant.

## Results

Overall, 30 SEA patients were included in our study, mostly women (n=18, 60.0%), with atopy (n=22, 73.3%), a mean age of 58.4 years (SD 11.7), and assisted by pulmonology (n=19, 63.3%) services. Patients’ mean eosinophilia at baseline was 1103.57 cells/μL (SD 604.88), ranging from 460.0 to 2400.0; half of them had severe eosinophilia (>900 cells/μL). After the use of benralizumab, the count of eosinophils dropped to zero (mean difference after six months: -1322.57; p<0.0001) (Table 1).

**Table 1:** Patients’ baseline characteristics (n=30)

Variable	N
Age, mean (SD), min-max	58.4 (SD 11.7), min-max 31.0-81.0
Sex	
Male	12 (40.0%)
Female	18 (60.0%)
Atopy	
Yes	22 (73.3%)
No	8 (26.7%)
Assistance service	
Pulmonology	19 (63.3%)
Allergology	11 (36.7%)
Eosinophils count cells/μL, mean (SD), min-max	1130.6 (SD 604.9), min-max 460.0-2400.0
Severe eosinophilia (>900 cells/μL)	
Yes	15 (50.0%)
No	15 (50.0%)

**Figure 1:**
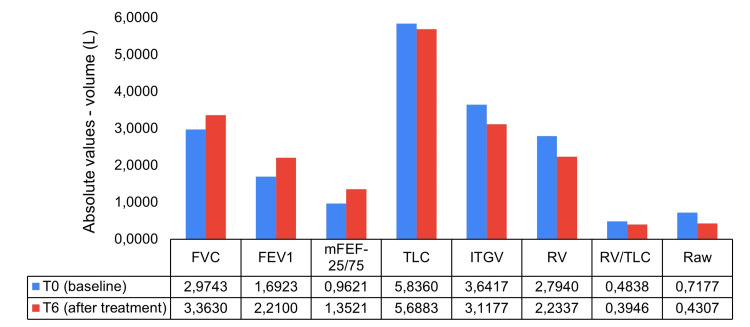
Differences in static lung volumes and airway resistance measures six months after benralizumab: absolute values FEV_1_: forced expiratory volume in one second (liters); FVC: forced vital capacity (liters); ITGV: intrathoracic gas volume (liters); mFEF-25/75: mean forced expiratory flow between 25% and 75% of FVC (liters/second); Raw: respiratory airway resistance (kPa*s/L); RV: residual volume (liters); TLC: total lung capacity (liters)

Absolute differences in static lung volumes and airway resistance measures after benralizumab treatment are depicted in Figure 1.

**Figure 2:**
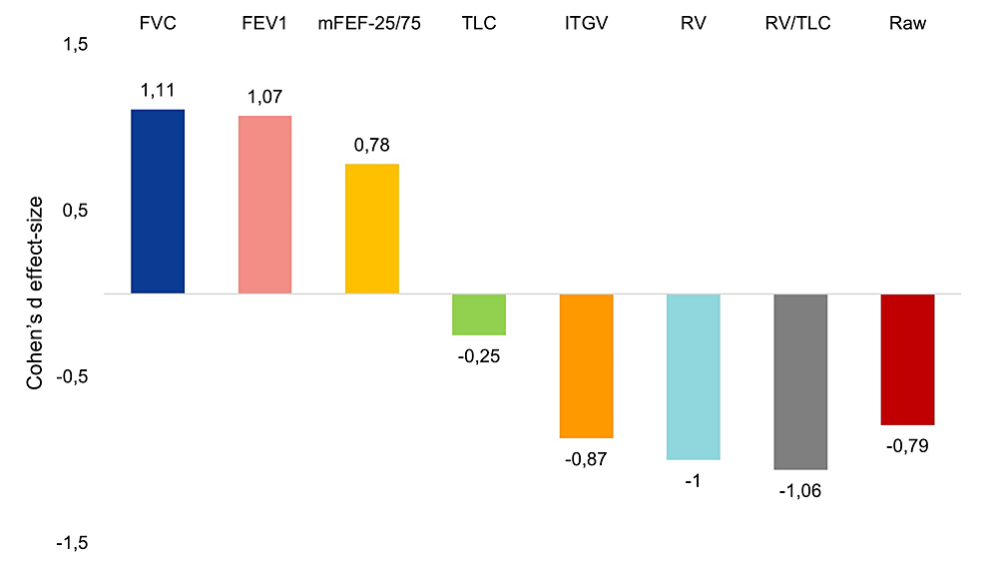
Differences in static lung volumes and airway resistance measures six months after benralizumab: Cohen’s d effect size FEV_1_: forced expiratory volume in one second; FVC: forced vital capacity; ITGV: intrathoracic gas volume; mFEF-25/75: mean forced expiratory flow between 25% and 75% of FVC; Raw: respiratory airway resistance; RV: residual volume; TLC: total lung capacity

Significant increases in FVC (pp 15.3%; p<0.0001), FEV_1_ (22.6%; p<0.0001), and mFEF-25/75 (17.7%; p<0.0001) were observed from baseline, with Cohen’s d large effect sizes of 1.11, 1.07, and 0.78, respectively (Figure 2). Conversely, the use of benralizumab led to significant decreases in ITGV (pp -17.3%; p<0.0001), RV (-29.7%; p<0.0001), RV/TLC (-8.9%; p<0.0001), and Raw (-100.6%; p<0.0001), also with large effect sizes (d between -0.79 and -1.06). No meaningful differences in TLC after treatment were obtained (pp -2.3%; p=0.173; small effect size: d=-0.25).

Measures were similar between patients according to sex, presence of atopy, and eosinophilia severity (p>0.05), except for FEV_1_ and mFEF-25/75, where patients with more significant eosinophilia (>900 cells/μL) presented better responses (p=0.001 and p=0.007; respectively) (Table 2). No adverse effects were recorded during the study period.

**Table 2:** Results by sex, atopy, and eosinophils count (differences in pp) FEV_1_: forced expiratory volume in one second; FVC: forced vital capacity; ITGV: intrathoracic gas volume; mFEF-25/75: mean forced expiratory flow between 25% and 75% of FVC; Raw: respiratory airway resistance; RV: residual volume; TLC: total lung capacity; pp: percentage points *Paired t-test (two-sample t-test with equal variances)

Measure	Sex variable			Atopy			Eosinophilia		
	Male (n=12)	Female (n=18)	p-value*	Yes (n=22)	No (n=8)	p-value*	<900 cells/μL (n=15)	>900 cells/μL (n=15)	p-value*
FVC	12.5	17.2	0.185	13.9	19.5	0.839	12.5	18.2	0.138
FEV1	25.7	20.5	0.740	22.7	22.5	0.491	11.5	33.8	0.001
mFEF-25/75	34.3	35.8	0.400	34.9	36.0	0.566	28.7	42.0	0.007
TLC	-106.2	-108.6	0.680	-109.4	-102.7	0.886	-108.4	-110.4	0.172
ITGV	-127.7	-123.3	0.334	-127.4	-119.2	0.781	-124.2	-126.3	0.588
RV	-93.1	-109.1	0.809	-102.5	-101.7	0.515	-107.8	-96.9	0.284
RV/TLC	-41.3	-41.9	0.966	-45.5	-44.9	0.554	-44.7	-45.8	0.615
Raw	-107.9	-144.6	0.712	-145.7	86.6	0.792	-136.5	-123.4	0.419

## Discussion

In the context of our multicentric observational study, benralizumab significantly improved respiratory function in patients with SEA, both atopic and non-atopic, after six months of treatment. This improvement was particularly notable in static lung volumes, airflow, and airway resistance. The therapeutic effect is likely due to the eosinophil depletion induced by benralizumab, as high blood eosinophil levels are associated with severe airflow limitation [1-3].

Compared to baseline values, benralizumab treatment resulted in an average increase in FEV_1_ of 520 mL after six months, exceeding the highest increases observed in the SIROCCO, CALIMA, and BISE trials (159 mL, 125 mL, and 80 mL, respectively) [21,22,26].

Our results were similar to those found by Pelaia et al. (2020), where benralizumab increased by 505 mL and 690 mL after four weeks and 24 weeks of treatment, respectively [13]. FVC also increased by 389 mL after six months of treatment. Both FEV_1_ and FVC changes showed large effect sizes, with Cohen’s d values of 1.11 and 1.07, respectively.

No significant differences were observed in the measures based on sex, atopy presence, and eosinophilia severity (p>0.05), except for FEV_1_ and mFEF-25/75. Patients with higher eosinophilia levels (>900 cells/μL) exhibited more favorable responses (p=0.001 and p=0.007, respectively) to these specific parameters.

Even more importantly, for patients with SEA and air trapping, our results also showed that, in comparison to baseline median values, benralizumab induced significant decreases of the ITGV, RV, and RV/TLC (524 mL, 560 mL, and 8.9%, respectively), with large effect sizes (Cohen’s d between -0.79 and -1.06). These clinically meaningful deflating effects are consistent with those reported by Pelaia et al. (2020) and by the SOLANA trial [13,25], indicating an effective pharmacologic action on the small airways, improving expiratory flow, and reducing air trapping. In this regard, we found that, with respect to baseline, mFEF-25/75 increased by 390 mL/s after six months of treatment with benralizumab (17.7%; Cohen’s d=0,78). Similarly, our results showed that benralizumab significantly reduced airway resistance.

Eosinophils play a pivotal role in the airways of asthmatic patients, contributing significantly to the pathophysiology of asthma, namely, (a) release of several molecules, such as leukotrienes, eosinophil peroxidase, and eosinophil cationic protein, contributing to airway inflammation and tissue damage; (b) tumor necrosis factor involved in the activation and recruitment of eosinophils, contributing to the inflammatory response in asthma and transforming growth factor-beta that can promote tissue remodeling and fibrosis in the airway; (c) eosinophils interacting with goblet cells in the airway epithelium, leading to increased production and release of mucus; (d) eosinophils releasing various mediators that can induce contraction of smooth muscle cells in the airways that further contributes to airway narrowing and increased resistance to airflow; and (e) the release of toxic granule proteins and other inflammatory mediators by eosinophils that can cause direct damage to the airway epithelium, leading to tissue injury and exacerbating the inflammatory response [4-6,13]. Our findings suggest that benralizumab may attenuate airway eosinophilic inflammation and bronchial remodeling, thereby improving airflow limitation, lung hyperinflation, and reducing asthma exacerbations.

Out of the participants enrolled in this real-world study, 22 were identified as having allergies. Our results corroborate the previously reported effectiveness of benralizumab in both atopic and non-atopic subjects with SEA [4-8].

The main limitations of this study are the relatively small number of recruited patients, a brief observation period of six months, and, as with many real-world investigations, the absence of a placebo control. However, our results suggested that benralizumab's suppression of eosinophilic inflammation can improve airflow limitation and lung hyperinflation.

## Conclusions

The eosinophil depletion with add-on benralizumab led to notable improvements in SEA patients’ respiratory function, namely, static lung volumes, airflow, and airway resistance, in real-world settings after six months of treatment. The significant deflating effect of benralizumab on hyperinflated lungs was evidenced by expedited expiratory flow (increased FEV_1_ and mFEF-25/75) and reduced air trapping (decreased RV/TLC).

Collectively, our results indicate that benralizumab improves bronchial obstruction, lung hyperinflation, and airway resistance. Based on these findings, it is possible to consider this anti-IL-5Rα monoclonal antibody as a potentially valuable therapeutic option for SEA patients, such as those enrolled in our study. These real-world preliminary findings need to be further validated by pragmatic trials that should be performed in larger cohorts of SEA patients and with a longer follow-up period.
